# How Sex Workers Understand Their Experiences of Working in the Republic of Ireland

**DOI:** 10.1007/s13178-021-00626-2

**Published:** 2021-08-03

**Authors:** Adeline Berry, Patricia Frazer

**Affiliations:** 1 Department of Health and Human Sciences, University of Huddersfield, Queensgate, HD13DH Huddersfield, UK; 2 Department of Psychology and Social Science, Dublin Business School, Dublin, Ireland

**Keywords:** Sex work, Client criminalisation, Brothel keeping, Ireland, Republic of Ireland, Policing, Stigma

## Abstract

**Introduction:**

This study seeks to explore the ways in which sex workers understand their experiences of working under sex work legislation in the Republic of Ireland, including laws that criminalise the purchase of sexual services. Participants reflected on their experiences of working in Ireland both and after the passing of [the] Criminal Law (Sexual Offences) Act 2017. In 2017, the Republic of Ireland criminalised the purchase of sexual services and increased fines and sentences for brothel keeping.

**Method:**

In 2020, semi-structured interviews lasting 60 to 90 min were conducted with 6 sex workers from diverse backgrounds, ages 24–44, actively working in Ireland since 2017. Interviews were recorded and transcribed verbatim. Transcriptions were used to conduct an interpretative phenomenological analysis.

**Results:**

Seven themes arose from the data: psychological wellbeing, relationships with law enforcement, relationships with friends and family, the effects of client criminalisation laws on clients, benefits of sex work community, stress related to precarious accommodation and experiences of both discrimination and perceived discrimination.

**Conclusion:**

Changes to sex work legislation appear to have failed in their mission to improve life for sex workers in Ireland. Other options such as decriminalisation should be considered.

In the Republic of Ireland, sex work itself is not illegal, but many aspects surrounding sex work are criminalised, such as workers working in the same premises together (Sexual Offences, 1993). In 2017, the purchase of sexual services was criminalised, and penalties and sentences for brothel keeping were increased (Sexual Offences, 2017). Gathering accurate data for numbers of sex workers in the Republic of Ireland is difficult due to them being a hidden population, often because of cultural stigma and fear of legal repercussions (Ellison et al., 2019; Huschke & Ward, 2017; O’Connor et al., 1996; Sweeney & FitzGerald, 2017). The majority of sex workers in Ireland, such as escorts or dominatrixes, work indoors; while a small percentage, street workers, work outdoors. No typical profile is available, as sex workers themselves are as diverse as their experiences and the paths that led them to sex work; however, many engage in sex work for reasons such as poverty and inequality (O’Connor et al., 1996; Valiulis et al., 2007).

In the 1920s, the new government of the Irish Free State, heavily influenced by the Church, began the process of enshrining Catholic morality into law, censoring anything perceived to negatively influence sexual conduct, from alcohol to publications, film and dance halls (Crowley & Kitchin, 2008). In 1935, under a Fianna Fáil government, [the] Criminal Law Amendment Act, 1885, was amended to criminalise brothel keeping, while penalties for engagement in sex work were increased (Amendment Act, 1935, 13). With the cooperation of the state, charitable institutions founded in the 1700s to house and rehabilitate ‘fallen’ women became prisons for sex workers and the unmarried pregnant daughters of Catholic families. From the 1930s onward, the state paid religious orders such as the Sisters of the Good Shepard, the Religious Sisters of Charity and the Religious Sisters of Mercy capitation grants to house ‘problem girls’ (Smith, 2010, 3). Escapees from these institutions would be returned by the Gardaí Síochána, the police force in the Republic of Ireland. ‘If the Gardaí managed to find the escapees, there was a consistent practice of returning them to the Magdalene Laundries where they suffered punishments ranging from solitary confinement, deprivation of meals and the shaming and humiliating practice of hair cutting’ (Smith et al., 2013, 82).

Following efforts by historian Catherine Corless, the discovery of a mass grave containing the bodies of 796 children was discovered in 2014 at the Bon Secours Mother and Baby Home in Tuam, Galway. This signalled the beginning of the end of the Magdalene Laundries and Mother and Baby Homes. The scandal attracted worldwide attention, inspiring a popular and award-winning film ‘the Magdalene Sisters’ (Mullan et al., 2002). While the last of the Mother and Baby Homes closed in 2006 (Hogan, 2019), the religious orders that ran them continue to sit on the boards of influential non-governmental organisations, and as of this article, there is contention as to the role these religious orders will play in the running of Ireland’s new national maternity hospital (Lynott, 2021).

The Oireachtas is the major governing body in the Republic of Ireland and is composed of two houses: Dáil Éireann and Seanad Éireann. The Oireachtas is headed by the president but led by the Taoiseach or prime minister. The Dáil is the more powerful of the two houses and composed of Teachta Dála or TDs, elected by the public, while members of the Seanad, Senators or Seanadóirí are nominated by the Taoiseach, politicians and universities. Bills must be approved by the Dáil but are also sometimes approved by the Seanad which has the power to delay but not veto bills. While the Republic of Ireland has several political parties, the two most powerful parties to date have been Fianna Fáil, founded in 1926, and Fianna Gael, founded in 1933.

Although the primary purpose of the law was to decriminalise homosexuality following a ruling at the European Court of Human Rights (McDonagh, 2017), [the] Criminal Law (Sexual Offences) Act 1993 implemented a number of changes to sex work legislation. The legislation was introduced to criminalise both the organisation of sex work, which means controlling or directing the activities of one or more sex workers and living off the earnings of a sex worker as well as loitering for the intention of supplying sexual services. Search powers for Gardaí (police) and fines for providing false information were increased along with fines and sentences for soliciting and brothel keeping. Brothel keeping can include being a landlord, manager, tenant or occupier of a building while knowingly permitting any part of the premises to be used for sex work (Sexual Offences, 1993). As [the] Criminal Law (Sexual Offences) Act 1993 criminalises knowingly renting to sex workers, this can make renting property difficult for sex workers, contributing to a precarious housing situation. Additionally, the law criminalises sex workers working together for reasons such as safety and potentially criminalises sex workers living with roommates, partners or family, especially if the sex worker is contributing to rent or utilities. Amnesty International (2016) advises against passing laws that prohibit organisational aspects of sex work such as renting secure premises, working together, hiring security or other support staff, as these laws penalize workers for attempting to operate in safety, denying their right to security of person.

In 2009, Turn Off the Red Light (TORL), a campaign composed of more than 60 influential non-governmental organisations, was founded in the Republic of Ireland with the intention of eradicating sex work (TORL, 2017; Huschke & Ward, 2017; McGarry & FitzGerald, 2019). TORL, the driving force to implement client criminalisation in Ireland, was spearheaded by Ruhama, the Immigrant Council of Ireland and the Religious Sisters of Charity. Ruhama is a state-funded organisation based in the Republic of Ireland that works with sex workers (Flynn, 2016), while the Immigrant Council of Ireland is a state-funded organisation that works with migrants. Immigrant Council of Ireland was founded by Sister Stanislaus Kennedy of the Religious Sisters of Charity, while Ruhama was founded by the Religious Sisters of Charity and the Good Shepard Sisters.

In 2009, the Immigrant Council of Ireland published a report ‘Globalisation, Sex Trafficking and Prostitution: The Experiences of Migrant Women in Ireland’ (Kelleher et al., 2009). In the foreword, Sister Stanislaus Kennedy states, ‘there is no clear line between where the elements of trafficking end and “consent” to become involved in the sex industry begins’. Article 3(a) of the Palermo Protocol (Annex, 2000) is employed to defend this obfuscation of the word ‘consent’ throughout the study. The article states that trafficking in persons shall mean the.

*use of force or other forms of coercion, of abduction, of fraud, of deception, of the abuse of power or of a position of vulnerability or of the giving or receiving of payments or benefits to achieve the consent of a person having control over another person, for the purpose of exploitation (Annex,* 2000*).*

The report details that many women engaged in sex work are vulnerable due to extreme poverty, multiple forms of discrimination, disadvantage, abuse and life circumstances and therefore ‘had no real choice’ (Kelleher et al., 2009, 1).

*The Swedish legislation (client criminalisation) aims to eliminate all forms of prostitution, based on the premise that consent and force are difficult to determine, and that prostitution and trafficking cannot be separated. In this perspective, working in prostitution is viewed as being the result of a lack of choice and, for this reason, cannot always be regarded as being voluntary, since it is the result of deep structural inequalities, poverty and the lack of agency of women (Kelleher et al.,* 2009*, 155).*

The report concludes that rather than tackling inequalities that leave individuals vulnerable and relying on sex work, the priority must instead lie with criminalising the men that purchase sexual services, as ‘Prostitution is about male sexual power. Without male demand, prostitution would not exist’ (Kelleher et al., 2009, 39). While acknowledging that workers may enter sex work due to a lack of preferable opportunities, the report fails to consider how removing sex work might affect sex work without them first being offered preferable alternatives.

‘Carceral feminism’ is defined as a reliance on the power of the state to resolve and address inequality and violence against women and marginalised communities (Bernstein, 2010) while overlooking the role of the state in the creation and maintenance of violence and inequality. Weitzer (2007) detailed the ways in which anti-sex work activists often portray clients as ‘folk devils’. This is evident in the language used throughout the TORL report, for example, ‘Themes of perversions proliferate, particularly those of acting out fantasies by buyers who associate sex with filth and bodily fluids’ (Kelleher et al., 2009, 90). In the report, accounts of sex workers ‘sound like some mysterious nightmare’ (Kelleher et al., 2009, 54) where ‘voodoo is used in the recruitment process and in subsequently psychologically enslaving young women and keeping them in the grip of traffickers’ (Kelleher et al., 2009, 64).

The voices of those most affected were excluded from debates on changes to sex work legislation. As there is little literature supporting client criminalisation, voices from influential members of Irish society have been included instead from debates leading up to changes in sex work legislation. During the 2012 Oireachtas review of Irish legislation regarding sex work, it was acknowledged by Vanessa Hetherington of the Irish Medical Organisation that sex work exists in Ireland as a ‘consequence of the range of inequalities that women face in Irish society, in particular socioeconomic inequalities, barriers to active participation and inequality of opportunities and outcomes’ (2012). Denise Charlton of The Immigrant Council of Ireland claimed that ‘some of the research and evaluation from the Swedish model (client criminalisation) indicated women feel safer in the Swedish context (client criminalisation) than they did prior to the introduction of the legislation’ (2012). After a visit to Sweden, Senator Ivana Bacik stated that.

*We were impressed not only by the enormous benefit it has provided to former sex workers, who had been the sellers of sex, but also to the positive good in society. The main point is that the Swedish approach (client criminalisation) protects sellers of sex - the sex workers - from the harm and exploitation that many people who work in the front line say is inherent in the act of prostitution (*2013*).*

The HSE (Health Service Executive) is the publicly funded body responsible for the provision of health services in the Republic of Ireland. Ms. Linda Latham (2013), a clinical nurse with extensive experience in the field of women’s health as manager of the HSE’s women’s health services stated,

*It has been suggested that if prostitution were decriminalised for women and the purchaser of sex were criminalised, that would make it harder for women. I believe the opposite. By decriminalising women, we will be relieving them of the pressure they are now under to be covert, and they will not face court cases for prostitution-related offences as they do now. They would be encouraged to report violence, robberies and rape without fear of retribution or further incriminating themselves. They would not face fines, imprisonment or deportation, and would be able to access services such as women's health services and obtain full support to exit prostitution.*

In a Dáil debate, Deputy Thomas Pringle (2013) suggested that ‘Prostitutes would be safer under this legislation because they would be the victims, and thereby could report acts of violence and seek medical treatment without ramification. Once people accept that prostitution is a barrier to gender equality and a form of violence against women, their opinion changes’. Evidence from Sweden, however, contradicts statements by Irish supporters for client criminalisation. Client criminalisation, also known as the Nordic or Swedish model, originated in Sweden in 1999 (Danna, 2012) and is form legislation that targets the clients of sex workers rather than the sex workers themselves. The client criminalisation model of sex work legislation has been adopted by Norway, Iceland, Canada, Northern Ireland, France and Israel. Trafficking and sex work still exist in Sweden despite having client criminalisation since 1999, with resources being directed toward the pursuit of sex worker clients rather than tackling more serious criminal activity (Huschke & Ward, 2017). Sex workers in Sweden are routinely evicted from hotels and their places of business (Levy, 2014). In one 2-year study that included 113 interviews with sex workers from Sweden, Norway and Finland, some felt the power dynamic had been reversed under client criminalisation, with the worker now focused on making their client feel safe (Vuolajärvi, 2019). Sex workers in Sweden have reported increased competition due to a reduction in clients leading to desperation, rushed negotiations and willingness to engage in riskier acts (Levy, 2014). High levels of stigma and increased marginalisation are reported (Levy, 2014), with poor conditions exacerbated by the lack of support services for sex workers, including those that wish to transition out of sex work (Levy & Jakobsson, 2014). While some nationals in countries where client criminalisation is in place felt they could avail of exit supports, migrant workers were excluded, facing eviction and deportation instead despite the selling of sexual services being legal (Levy, 2014; Vuolajärvi, 2019).

Research in Northern Ireland found that rather than reducing numbers engaged in sex work as per the law’s intention, the industry has grown in size, with higher numbers providing than before the law, with little reduction in numbers of clients and little change in habits relating to the purchase of sexual services. Numbers of reported robberies and assaults against sex workers were shown to have increased under the law, with no decrease noted in the number of trafficking victims (Ellison et al., 2019). Amnesty International (2016) stressed that in order for states to protect sex workers from harm, states must avoid passing laws that compromise the safety of workers by leading workers to take risks to protect their clients, such as agreeing to meet clients in more dangerous locations to avoid detection by law enforcement, which is likely under a model that criminalises the clients sex workers depend on for income.

In March 2017, the government of the Republic of Ireland under Fine Gael leadership implemented [the] Criminal Law (Sexual Offences) Act 2017 which amended [the] Criminal Law (Sexual Offences) Act 1993 by criminalising the purchase of sexual services, instituting a fine of up to £1000. Sentences were doubled for brothel keeping, organisation of sex work and living off the earnings of a sex worker, while fines for each were increased from £1000 to a maximum of £5000. Considering the purpose of the law is to penalise the client rather than the worker (Latham, 2013), it is worth noting that while there is no jail sentence for clients, workers who work together for safety are at risk of imprisonment and face considerably larger fines. In 2019, 2 Romanian women aged 23 and 25, one of them pregnant, were sentenced to 9 months in prison in the Republic of Ireland by following a raid by Gardaí (police) despite there not being clients or a significant amount of money present (Lynott, 2019).

In 1995, 86 sex workers in the Republic of Ireland were interviewed by peer interviewers as part of a study commissioned by the Women’s Health Project as part of the European EUROPAP PROJECT, a programme to tackle HIV/AIDS amongst sex workers (O’Connor et al., 1996, 26). The research found that 1993 changes to sex work legislation in the Republic of Ireland were putting women’s lives at risk. Additionally, participants perceived most Gardaí (police) to be abusive, displaying disregard for the feelings of the workers.

*The new legislation has had an impact on the working lives of most of the women interviewed. For many, work has become much more stressful; constantly being moved on; working in fear of raids and of their identities being exposed. For the majority of the women interviewed the new legislation has meant a decline in their working conditions. Because they are constantly being moved on by the Gardai they are having to work longer hours to make the same amount of money as previously. This has contributed to making working conditions more dangerous, with the increased pressure to maintain a certain level of income leading to greater risk taking on the part of the women. Risks are also being taken in relation to choice of clients in that women are getting into cars more quickly with no time to study prospective clients, with implications also regarding risk taking in relation to HIV/AIDS (O’Connor et al.,* 1996*, 26).*

A 2007 study commissioned by the Irish Human Rights Commission (Valiulis et al.) reported that sex workers in Ireland were likely to avoid contacting emergency services following an assault. The study included interviews with 7 Irish participants with a history of sex work. Research that looked at the psychosocial experiences of migrant sex working women in Ireland (Sweeney & FitzGerald, 2017), conducting interviews with 19 workers across Ireland, found that migrant sex workers faced discrimination from the public and were reluctant to contact authorities even in the event of being stolen from or raped because of language difficulties and for fear of jeopardising their visas or status as asylum seekers. In a meta-analysis of 40 quantitative studies and 94 qualitative studies published between 1990 and 2018, Platt et al. (2018) found policies that criminalise sex work discourage workers from accessing adequate healthcare and act as a barrier to accessing social care.

Sex workers in Ireland have reported relationship problems and inabilities to form emotional connections (Valiulis et al., 2007), though how rates of relationship problems and intimacy issues amongst sex workers compare to those of the general populace are not mentioned. Stigma (Goffman, 2009) has been defined as any physical or social attribute or sign that so devalues an actor’s social identity as to disqualify him or her from full social acceptance. Sex workers report reluctance to disclose their occupation to current or future partners due to shame and stigma, which compels them to live double lives (Valiulis et al., 2007), fabricating stories to tell friends and family in order to avoid marginalisation (O’Connor et al., 1996; Sanders, 2004; Valiulis et al., 2007), which can be a source of considerable stress in itself (Di Marco et al., 2020). Sex workers have described hostility and harassment from community, services and Gardaí as sources of stress and stigma (Valiulis et al., 2007). UglyMugs.ie is a mobile phone app and website where sex workers in Ireland can record negative interactions with clients. An analysis of UglyMugs.ie data from 2015 to 2019 (Campbell et al., 2020) found that violence against sex workers increased in the period following the implementation of client criminalisation but that reporting to Gardaí (police) remained low.

## Research Aims and Rationale

At the time of writing, there has been very little qualitative research into the experiences of sex workers published in the Republic of Ireland since the implementation of the Criminal Law (Sexual Offences) Act 2017, exploring the effect of the law on the lives of sex workers. Sex workers are often spoken about and legislated for without being included in the conversations concerning them (McGarry & FitzGerald, 2019). It is the intention of this research to elevate the voices of those directly affected by sex work legislation. A review of [the] Criminal Law (Sexual Offences) Act (2017) after 3 years by the Justice Department was written into the legislation, scheduled for March of 2020. At the time of writing, the review process is under way, considerably delayed by the COVID-19 pandemic. It is the aim of this study to understand how sex workers in the Republic of Ireland understand their own experiences of working under current legislation.

## Methods

### Participants

In accordance with Smith and Osborn (2015), 6 sex workers actively working in Ireland since 2017 were selected as participants due to their experience of working under the Criminal Law (Sexual Offences) Act (2017). The age range was 24 to 44 (*M* = 30 years old; *SD* = 6.66 years old). Four sex workers reported their gender as cisgender female, and two identified themselves as non-binary. Three participants reported their nationalities as Irish, while the remaining participants reported their nationalities as British, Croatian and Brazilian. One participant identified themselves as an outdoor sex worker, one as a dominatrix, three as full-service escorts and one as a full-service and online worker. All participant names have been anonymised. All participants worked throughout Ireland. One participant was based in Northern Ireland, while the remainder were based in Dublin. All participants were recruited through convenience sampling.

### Data Collection

The aim of this research was to learn from sex workers themselves how they understand their experience of working under the Swedish model of law enforcement. The interview schedule was influenced by Shinebourne and Smith (2009) and altered to suit the requirements of the current research. It featured questions such as ‘How does being a sex worker in Ireland affect your life?’ designed to elicit from the participant reflections on their circumstances, with ‘How do you think being engaged in sex work elsewhere might be different? What different choices might you have made if you lived somewhere else?’ serving as possible prompts if necessary. Interviews were conducted in locations of the participants’ choosing. Two interviews were recorded in isolated corners of public houses, two were recorded in the homes of participants and two were recorded in the home of the lead author. All interviews were conducted by the lead author. Interviews lasted from 30 min to an hour. Informed consent was obtained from all participants who were informed of their right to withdraw from the research and supplied with mental health resources. No incentives, financial or otherwise, were offered.

### Analysis

Sex workers are often silenced by stigma and fear, so it is important that an approach be employed that allows for an exploration of the ways in which sex workers made sense of their own realities, allowing their voices to be heard. Phenomenological research is concerned with perception and the meaning people attribute to their existences. Interpretative phenomenological analysis (IPA) combines phenomenology and hermeneutics, building on the work of Husserl, Heidegger, Schleiermacher, Gadamer and others, to uncover the hidden meaning behind a person’s words and to explore how people make sense of, and find meaning in, their own lived experiences.

*IPA is committed to understanding how particular experiential phenomena (an event, process or relationship) have been understood from the perspective of particular people, in a particular context. As a consequence, IPA utilises small, purposively selected and carefully situated samples, and may often make very effective use of single case analyses (Smith et al.,* 2009*).*

Other forms of qualitative analysis were considered. As this project is more concerned with elevating the voices of sex workers in Ireland than generating theory, the Grounded theory was not used. As this research was more interested in elevating the content of what sex workers had to say rather than how they said it, narrative analysis was ruled out. This research worked with a small but diverse sample and was interested not just in what the participants had in common but also where their experiences diverged, so thematic analysis was not chosen as a method of analysis. This research is interested in how sex workers in the Republic of Ireland experience client criminalisation. As ‘IPA is usually concerned with experience which is of particular moment or experience to the person’ (Smith et al., 2009), it is particularly suited to the subject.

## Results

Participants recounted their experiences of sex work in the Republic of Ireland, sharing their reflections on community, policing, mental health, relationships and the cultural environment in which they work. Some shared their experiences of engaging in sex work in countries and how it compared to working in Ireland. Most relevant for this research, the majority of participants conveyed their perception of working in Ireland both before and since the implementation of client criminalisation. This analysis detailed a number of recurrent themes. In ascending order, the themes that arose most often were mental health and policing; family and friends and sex worker community; clients; housing, security and stability; and discrimination.

### Psychological Wellbeing

This section is included in part because the theme arose amongst participants so frequently and also because it helps paint a picture of the landscape in which these workers exist and the culture in which these laws are written and implemented. Participants discussed a complex range of mental health states, ranging from fear of Gardaí (police) to feelings of powerlessness and isolation. Some participants discussed finding some sense of empowerment through entry into sex work, even if fleeting, while for one sex work sat within an existing cycle of trauma. A couple of participants related an improvement in self-image and feelings of self-worth as they overcame the stigma and shame associated with sex work. Sandra, an indoor sex worker, speaks here about first venturing into sex work in Florida, and the initial freedom she felt, liberated from what she perceives to be overwhelming pressures of Irish societal expectations.

*I absolutely loved it to begin with, but you know when you travel, you can sort of be a different person, like there’s no way I would have done it over here, but it’s amazing when you’re, when you’re away from…not having to worry about, what other people think, because that’s massive in Ireland, isn’t it? You, when, you know you’re stifled by what people will say….*

For Rachel, an indoor sex worker, entry into sex work facilitated a sense of empowerment and independence.

*You know, running, essentially running my own business, and, you know, at the end of a week looking at, or going to the bank to lodge my money, and thinking, god, you know, I was able to make this money with, essentially no help from anyone, you know, not working within a company, not even working out of a hotel, but just working from my own apartment. It’s given me a sense of… power and independence that, you know, I didn’t have before.*

Initially, Tara, an indoor sex worker, experienced an initial sense of empowerment and excitement from sex work before it gave way to a sense of mundanity.

*It definitely was, like, something that was like, that I thought was exciting, like, gave me all this empowerment, then became something that was kind of boring for me, and it was just something that I kinda have to do just in order to make money, and it’s more like a schlog (laughs).*

Rachel discusses the sense of independence a pride sex work gave them while allowing them to work around existing mental health issues.

*That sense of the independence that sex work gave me and the, that independence removing the reasons for my anxiety, my financial anxiety, and my anxieties about rejection if I was applying for a job, my anxiety about being fired from a job because of my mental health, you know, those things were all suddenly much easier to deal with, or in some cases removed entirely. So, I felt really, really positive, and I felt I suppose relief, and I don’t know, maybe a certain sense of accomplishment or pride or independence.*

Reflecting on her entry into sex work in the Republic of Ireland, Jade, an outdoor sex worker, tells a starkly contrasting story, as she still struggles to relate her feelings of powerlessness entering sex work for the first time, and how sex work for her fit within an existing cycle of addiction and homelessness.

*Working out of the flats, I had a very negative perception of what was happening, I did, I didn’t feel I had any control, and it was just … traumatic experience to me as you know, and that, that went along with the, the kind of wider circumstances of my life, you know, that I was either in care, or homeless, in addiction, and I really just felt powerless.*

Below Sandra expresses feelings of fear, isolation and otherness, struggling with the pressures of both stigma and internalised stigma. Her words help illustrate the wider cultural climate in which sex work, policing and legislation in Ireland are situated.

*But I’m pretty, pretty isolated. I don’t socialize much with people that aren’t sex workers really, I just don’t feel (laughs) comfortable, around it, …so you’re always kind of cowering in that fear…I’m feeling it there, I’m different, I’m a dirty secret, I’m, you, you know, are people looking at me and thinking that? So, it’s sorta, you definitely get para, paranoid, and I’m thinking my paranoia is quite justifiable considering, you know, everything that I have experienced, so, yeah, paranoia…because of how people…act, or…treat you or whatever.*

Jade relates how overcoming a sense of otherness and negative feelings toward sex work improved her sense of self and allowed her to embrace the positives and her strengths that grew from her experiences.

*I think I did a lot of work on myself to, to move… away from that place, but… seeing sex work differently, and seeing my place in sex work differently contributed to that. You know, so… not seeing… sex work as a, a kinda evil in my life, helped. …And also, not… seeing myself as just some dirty little street whore, yeah know, moving away from that and being able to see the strengths that, come from being a sex worker, and actually, a lot of the most positive aspects of me have some foundation in my experience as a sex worker, yeah know. So yeah.*

Tara reflects on the role of stigma in her life, assuring herself that she is not responsible for the stigma she endures nor the burden of living a double life. ‘I should not blame myself for the stigma around that, and I, I shouldn’t blame myself for the lying that I have to do because I’m doing it to protect myself’.

Sandra expresses fear and a feeling of otherness, as she describes the fear she senses within the sex working community in Ireland.

*Ireland at the minute, like because of the law changes feels really scary just now, especially being in groups where there’s supporting each other and sharing information, you can feel the fear of, from your, from the community, and you sort of feel like you’re a target and you don’t feel like I have the same rights as everyone else, I feel like I don’t have human rights*.

Jade expresses a feeling of overwhelming pressure as she ruminates on her own experiences and those of other street workers since the implementation of client criminalisation.

*It’s really just a feeling of being squeezed, of being overwhelmed and trying to just… get enough to, to get by because nobody wants to go and take a write off of a night, yeah know… One of the girls that is on crack and dying for a pipe, is, isn’t going to turn down that job, you know, because she can’t.*

### Policing

All participants, regardless of whether they were indoor or outdoor sex workers, Irish or migrant, consistently expressed negative perceptions of law enforcement. Some, like Sinead, express that policing is for them the worst part of the job. ‘I mean like, it’s just, it’s like, again, it’s not the job it’s the legality of it, right?’ One participant expressed cautious optimism for positive relations with Gardaí (police) under different circumstances, while another relayed how client criminalisation had been detrimental to her previously positive relationships with PSNI (Police Service of Northern Ireland). All participants expressed reluctance to contact law enforcement during an emergency. Sandra relates feelings of helplessness and a fear of being evicted by Gardaí again under brothel keeping laws. She relates how under current legislation hostility replaced what she felt was previously a positive relationship and how she no longer feels she can turn to law enforcement for protection in the current environment for herself and her family.

*I felt like the police were on my side three years ago because I’ve, I’ve had, had, I’ve had, had interactions with the police before and I’ve always felt like they were on my side if something was happening because of the, you know, the liaison officers [a member of the police force that acts as an intermediary between sex workers and police] and everything? Now it feels like, you know, nobody’s really on my side and I feel that, um, anyone who would seek to abuse me, they’re more empowered because of, of, of the fear of getting kicked out because people managed to successfully get me evicted, and threaten my kids and all that kind of stuff before, so I’ve gone through that and not had the, not had the police protection and fear of social services and all that, it’s like, it’s totally, it definitely feels completely different, and I felt more respected by police when you know they, they were telling me myself, when you’re working on your own out of your own home it’s a privacy thing, it’s not anything to do with us, but now it’s like, I dunno, it just feels a lot more, a lot more hostility?*

Rachel recounted how brothel keeping laws had contributed to their rape by a client and distrust in law enforcement had left them feeling as though they were without recourse.

*I thought, you know, there’s no one else in the apartment, I was having to work alone because of the laws around sex work in Ireland, and I thought it’s not safe for me to try and physically resist because if he becomes physically violent then I, I’m in a very dangerous situation. I don’t think I can protect myself, but my reaction to that again was to feel angry. First of all, angry at him. You know, slammed the door when he left, and kind of just… you know, felt physically at that moment, but I also felt angry that I couldn’t go to the guards about it. I felt angry that I’d had to work alone which had… you know, made it possible for him to do that. I felt angry that, I hadn’t been able to protect myself in that situation.*

Sinead reflects on her reluctance to contact law enforcement even in a life-or-death situation.

*I would probably never call them unless it was literally like, like life and death, like, like if I thought I was going to be killed I’m calling 911 but I think even then I might actually try to fight them off before I would call the police …. Like I would rather honestly be killed than call the cops, like there’s no question about that.*

Lucy, an indoor sex worker, ruminates on how her perception of relations with law enforcement changed after her entry into sex work. As a migrant worker, she feels a sense of heightened vulnerability.

*When it comes to interacting with the police, maybe that rea…that’s probably, I could point that as the worst part, because I have to be very careful when I tr, when I deal with, authorities, even if I am the victim of someone in the streets that, you know, every time I, I think of, what if I’m raped? I’m very scared of dealing with the police, so that changed. Because before, before being a sex worker, I could just come into any authority, and say this happened to me and I’m a victim, do something about it, but these days, I’m terrifying of them framing me instead of the man, for whatever happens to me. It affects my life because, I’m a migrant, and I have to be extra careful with everything I do to, avoid suffering legal consequences.*

Here, Rachel reflects on Gardaí (police) and their dual roles as both protector and persecutor and negative perceptions of Gardaí amongst the sex work community. They make suggestions for the provision of help for sex workers by the state without exacerbating conditions for workers or worsening relationships between sex workers and Gardaí.

*While the Gardaí are arresting sex workers, and deporting sex workers, and locking them up for brothel keeping, that’s not going to change, but if, you know, if support was being offered to sex workers, or, or sex workers, if the first point of contact between the state and sex workers was more often social workers, or … you know, the, if you were running a brothel and inspections were being carried out, inspections had to be carried out, excuse me, carried out by the health and safety authority rather than the Gardaí, that would be ideal I think, because the Gardaí are a vector of violence and discrimination and fear for sex workers.*

Jade suggests that her experience of law enforcement has never been positive, as she considers both the direct and indirect effects of policing on outdoor sex workers.

*It’s like, simultaneously it’s changed so much and yet it’s exactly the same, yeah know, … but… the… the Garda presence out on the beat is never helpful. When they’re targeting the clients that just increases the tension in the area. And it, it increases the tension between the working girls so that people are turning on each other.*

Rachel expressed misgivings related to the role of the Gardaí (police) as providers of state support for sex workers. There is a sense of cautious optimism for the role of Gardaí in providing assistance to sex workers that appears to be on hold under current circumstances, perhaps both legal and cultural.

*I think finding ways to bring, when you have to provide support to sex workers from the state, trying to offer that support through avenues other than the Gardaí whenever possible, because the Gardaí are often, you know, I think Gardaí make things worse for sex workers more often than they make things better at the moment unfortunately.*

### Relationships

Most participants described having to lead a double life to hide their engagement in sex work from friends and family as something that negatively impacted them. Irish participants in particular expressed a fear of family finding out or the repercussions of them having found out. One participant had already been disowned by family members after being outed. Irish participants that had worked in countries other than Ireland remarked on the increased openness and reduced stigma they found there. Tara compares the levels of stigma in Ireland she has experienced to working in Germany and Australia where she recalls workers having open and honest relationships with even older family members without negative repercussions.

*When I was in Germany, working in Germany and in Australia, where they have like, more progressive laws around it, and there’s like, not as much stigma, where there’s a lot more people who were out to their families, or saying, particularly to older members of families that, like, “oh, I do sex work!” and it didn’t have, like, and that they didn’t have like a negative perception, and particularly amongst kinda older members and stuff like that, it was mad that they were like, more… progressive in their views, and whereas like, here in Ireland that’s definitely not the case, as well.*

Here, Lucy relates her fears for her reputation and future should her occupation be revealed. She affirms to herself that she has no reason to be ashamed but shifts suddenly from first person singular to second person singular, picturing the destruction to her reputation that being outed might bring.

*It’s terrible! It feels like… even though I know inside of me that I don’t have a reason to be ashamed of what I do, it feels like this terrible fear that if people get to know about it, they will, they will start thinking lower of you, and they might spread the word around and you’re going to become a joke to people.*

Tara struggles to express the negative impact of living a double life and the dishonesty she feels she must engage in to maintain relationships, not just amongst family but also amongst friends.

*I feel like I don’t always have like, the most honest relationships with people, and it makes it very hard to have an honest relationship with my family in particular, but also certain groups of friends and stuff, like, that as well, if I’m kind of engaging, I almost feel like I have to be lying, all the time, and I find that, like, really, really hard to deal with.*

Rachel too relates the stress of living a double life and being forced to hide their profession from family members. There is a sense of acceptance that revealing to their family how they provide for themselves would potentially carry devastating consequences, so they carry the burden of lying to maintain their relationship.

*The main negative impact is with my family where, I’ve come out to them about being trans but I’m still, I’m still in the closet about being a sex worker. I’ve no plans to ever come out to them about being a sex worker, and I’m having to, you know, tell them a series of lies and then try and make sure all my lies match about what I’m doing for work. And that can be, that can be stressful. I won’t lie. That is a negative impact.*

Sandra reflects on how anti-sex work sentiment and policing procedures contributed to her being outed which in turn led to her being disowned by her family.

*…there, there’s a negative side that we are now being exposed to these brothel keeping raids and the fact that it’s empowered these vigilante groups that out your details and out you to people and you know, being sort of, disowned by your family because people are vindictive you know, so, so that’s, yeah shits really bad. (Sighs).*

### Clients

There was a difference in responses amongst sex workers that worked outdoors and those that worked indoors. Both indoor and outdoor sex workers spoke about an erosion of boundaries, while migrant indoor sex workers reported an increase in abusive and disrespectful behaviour under client criminalisation. One participant reported that outdoor sex workers were fighting amongst themselves for fewer clients while indoor sex workers reported an increase in clients. One Irish indoor sex worker attributed the increase to men being more cautious about approaching migrant workers under laws that criminalise clients. Jade reflects on how a reduction in clients has led to increased anxiety, prices being lowered and boundaries being eroded.

*You’re competing for fewer and fewer clients, and… you, you start to, to get anxious that you’re not going to get your money tonight, and so then you’re… when, when clients come along you, you agree to drop your prices just that little bit and then you agree to do a little bit more than you wanted to do and you agree to go to a place that you’re not really comfortable going to that wasn’t your place, so your boundaries are just being eroded.*

Some indoor sex workers reported an increase in entitled behaviour from clients. Sinead perceives a decrease in respect in both communication and behaviour from clients since the implementation of client criminalisation legislation. She perceives that clients are aware of the hostile climate for workers and leverage it to erode worker boundaries and bargain for cheaper prices.

*Clients are bolder and the things they ask for and how they ask for them. Much more bold about asking for unprotected sex, much more bold for asking for cheaper prices, much bolder when, you know, with how they treat you, … so, because they know, like you want to keep your business, they know you’re not going to be calling the police. They know that shit. You know, more fear with attacks because this gives attackers like a nice way, an easy way for them to enter and attack us cause who wants to call the police so they can destroy your business. Nobody. I don’t.*

Lucy echoed Sinead’s sentiments relating to an increase in abusive behaviour from clients since changes to the law. ‘From, from the clients, yeah, from the clients. Absolutely. The abuse got much worse from the clients in the last few, in the last few years. And you could see that gradually changing. You could see the shift, yeah’.

Jade reflects on the power imbalance between sex workers and clients and how pressure often translates into increased pressure on workers.

*But they always thought they were going to be arrested and they will always use that to… you know, any … any… pressure you put on them they’re just gonna transfer to… the working girls, you know, so, they’ll just use that to their advantage, and see what they can gain from it. They did then, they do now.*

### Sex Work Community

All participants spoke of the positive impact of the sex worker community, other sex workers with whom they could share safety and work tips as well as camaraderie. Most discussed struggling with poor mental health prior to finding sex work community due to isolation, shame and stigma. This was more prominent amongst Irish workers than amongst non-Irish workers. Tara reflects on the isolation she experienced before finding community.

*I think it was really difficult when I didn’t know other people who did the work that I did, and I felt a lot of shame around it, and when I met other people from the community it made me feel a lot better about it, and I think that definitely has, like, made a difference … It’s really important for me to be supportive of other people because, like, that isolation really negatively affected my mental health because I just didn’t know anyone else, who was doing it.*

Sandra discusses the positive impact of the sex worker community and the value of having contact with other workers to turn to with work-related questions.

*I quite enjoyed working with other ladies because it was really helpful to be around other women, with, you know with questions, and like ‘is it normal? Is this, you know, what do I do about this situation?’ So, so that was really good.*

Similarly, Rachel reflects on how finding community led to her gaining access to valuable safety tips and advice from other workers.

*I tried to look up things online, advice for sex workers and how to keep themselves safe, in retrospect I, I probably didn’t do enough research in that, there was a lot I learned later on after talking to other sex workers, when I kind of, came across other sex workers in Dublin online and, and met with one in particular who gave me a lot of very good advice about how to keep myself safe, and introduced me to things like UglyMugs, the app for sex workers.*

Almost all participants related the value of having access to a community of sex workers. When asked about the impact of engagement in sex work on their relationships, Sinead responded, ‘dunno, it enriches them to be honest. I’ve never had better friends, I’ve never had more healthy relationships than I do now, you know’.

### Accommodation

All participants expressed a fear of being evicted from their homes or hotels they were working in. One participant had already been evicted by Gardaí (police) for engaging in sex work. Those that had worked in other countries compared their experiences with working in Ireland. Tara reflects positively on her experiences of renting in other countries compared to her fears of eviction in Ireland and how Irish brothel keeping laws work to contribute to precariousness during Ireland’s housing crisis.

*You didn’t have to worry about whether your neighbours were going to report you, cause even when you work in apartments here, you have to be like so careful in case, like, something would happen to your apartment, and it’s particularly more acute because the housing crisis, and landlords trying to find a reason to evict you, and being a sex worker is definitely a very good one (laughs) for, for them to get rid of you….*

Sinead expresses concerns about eviction from hotels due to brothel keeping laws and how fear of eviction adds to existing fears of Gardaí and clients. ‘…and so, I constantly have to be moving in and out of hotels and like, be worried about clients, and be worried about Gardaí’.

Having been evicted previously by Gardaí under the brothel keeping laws in the Republic of Ireland, Sandra expresses her fear of being evicted again. ‘You have to be very paranoid about where you live, I think that you know I’m gonna be outed, I’m gonna get kicked out again’.

### Discrimination

All sex workers, regardless of whether they worked indoors or outdoors, spoke of either direct experience of discrimination or perceived discrimination. Rachel reflects on experiences of accessing services and how anti-sex work attitudes translate into a disregard for autonomy.

*There’s always this sense that, you know… their first response is to try and get me out of sex work, rather than asking me what support work do I want, because yes, some people do need support, you know, they want to exit work and they need that support, but I would much prefer if the support was tailored to the person coming in and saying, ‘what support do you want? Do you want to exit sex work, or do you want support while continuing to do sex work?’*

Sinead echoes Rachel’s words, reflecting on experiences of encountering anti-sex work sentiment when trying to access healthcare.

*Like you either have to pay a lot of money for a private practitioner, or you have to go to the really shitty cheap places where people treat you like shit …they’re anti sex work, and um, yeah, and they’re constantly trying to paint, paint you as a trafficking victim, or you have to lie, and then it’s like ‘Oh why are you getting tested every three months?’ …But like we are always constantly being shat on for doing it.*

Table 1 contains quotes from participants reflecting on their experiences of working both before and after the passing of [the] Criminal Law (Sexual Offences) Act 2017 separated by theme.

**Table 1:** Experiences of sex workers pre- and post-Crim Law (SO) Act 2017

Themes	Before Crim Law (SO) Act 2017	After Crim Law (SO) Act 2017
Mental health	‘I feel better about myself, like yeah, like I used to, you know, I come from a very abusive childhood, I’ve only learned how to have better boundaries, while doing sex work, like the first time I told a client no I started crying, but he was okay with it, and I never had to, you know I never felt like I could say no, so…’ ‘So, my mental health issues didn’t start or got more accentuated after sex work, they always existed, and that comes from my personal life, you know, it was completely not related’	‘You’re always kind of cowering in that fear’ ‘Now it feels like, you know, nobody’s really on my side and I feel that, um, anyone who would seek to abuse me, they’re more empowered’ ‘And then… you know, so it, so it affects everybody in the area and then, just for me, I feel like, you know, you’re being squeezed’
Policing	‘I felt like the police were on my side three years ago because I’ve, I’ve had, had, I’ve had, had interactions with the police before and I’ve always felt like they were on my side if something was happening because of the, you know, the liaison officers and everything?’ ‘I felt more respected by police when you know they, they were telling me myself, when you’re working on your own out of your own home it’s a privacy thing, it’s not anything to do with us’ ‘There’s definitely a different energy in Ireland than, when you compare it to Edinburgh, things are more, there’s more fear over here’	‘Now it’s like, I dunno, it just feels a lot more, a lot more hostility?’ ‘You know the fact that we have people pretending to be guards and trying to blackmail us for free sex, you know? That pretty much says it all so, it’s much more fearful now. It’s like night and day’ ‘You know I’ve noticed the difference since I came back, and, and in some ways, you know, street work is kinda fundamentally the same really. It’s like simultaneously it’s changed so much and yet it’s exactly the same, yeah know, … but… the… the Garda presence out on the beat is never helpful’
Relationships	‘I think the only thing is my immediate family are here. Not my immediate family, my, my family, a lot of my relatives and stuff like that are here as well, and like, I have to be really kind of mindful around that sort of stuff, and like, that you literally, like, I feel like I could be out more as a worker, let’s say in Australia or something like that, and like far away from my family, but because I’m not, I have to be just that little bit more careful around others, and I think that’s like, a kind of really big thing’	‘I feel like I don’t always have like, the most honest relationships with people, and it makes it very hard to have an honest relationship with my family in particular, but also certain groups of friends and stuff, like, that as well, if I’m kind of engaging, I almost feel like I have to be lying, all the time, and I find that, like, really, really hard to deal with’ ‘I’ve no plans to ever come out to them about being a sex worker, and I’m having to, you know, tell them a series of lies and then try and make sure all my lies match about what I’m doing for work. And that can be, that can be stressful. I won’t lie. That is a negative impact’
Clients	‘But they always thought they were going to be arrested and they will always use that to… you know, any … any… pressure you put on them they’re just gonna transfer to… the working girls, you know, so, they’ll just use that to their advantage, and see what they can gain from it. They did then, they do now’ 'But the fact is like, on the street anyway clients always thought they were going to be arrested. You know the, the … so… I’m not sure the clients know that there’s been a change, not all of them. It’s just if they see the guards out, they’re gonna scarper pretty quick.' ‘I mean really they’re pretty much the same. The, the ones that, there’s always going to be the ones that will try and … abuse any advantage that they might have, and they existed then, they exist now, but this is giving them an added … position of power to abuse, you know?’	‘Abuse got much worse from the clients in the last few, in the last few years. And you could see that gradually changing. You could see the shift, yeah’ ‘You’re competing for fewer and fewer clients, and… you, you start to, to get anxious that you’re not going to get your money tonight, and so then you’re… when, when clients come along you, you agree to drop your prices just that little bit and then you agree to do a little bit more than you wanted to do and you agree to go to a place that you’re not really comfortable going to that wasn’t your place, so your boundaries are just being eroded’ ‘Just clients are bolder. Clients are bolder and the things they ask for and how they ask for them. Much more bold about asking for unprotected sex, much more bold for asking for cheaper prices, much bolder when, you know, with how they treat you, ….’
Sex worker community	‘I quite enjoyed working with other ladies because it was really helpful to be around other women, with, you know with questions, and like “is it normal? Is this, you know, what do I do about this situation?” So, so that was really good’ ‘I think it was really difficult when I didn’t know other people who did the work that I did, and I felt a lot of shame around it, and when I met other people from the community it made me feel a lot better about it, and I think that definitely has, like, made a difference, that I sometimes, like, even things, just someone, like I often feel like I, I sometimes do like creative things, and there’s a whole thing about being honest and up front when you’re doing the creative processes and I’m doing it with people who don’t do, understand sex work, or may have like, negative perceptions around it, and I’m like, I can never truly with these people be completely honest and stuff’	‘I’ve never had better friends, I’ve never had more healthy relationships than I do now, you know’ ‘Ireland at the minute, like because of the law changes feels really scary just now, especially being in groups where there’s supporting each other and sharing information, you can feel the fear of, from your, from the community’ ‘And so, it created distance in a sense that we are terrified of working together, and maybe even moments like where I could be working with other girls, because we would make more money in that way, it’s not possible anymore’ ‘It changed how… I interact with other workers because I cannot work with them anymore’ ‘I think it was really difficult when I didn’t know other people who did the work that I did, and I felt a lot of shame around it, and when I met other people from the community it made me feel a lot better about it, and I think that definitely has, like, made a difference’
Accommodation	‘Like, I think the only thing is when I was in Australia, like, it was mad, you would work in hotels and they literally did not give a fucking shit, at all, a lot of them, like, they kinda knew what you were up to and it was just like, “just go on,” or even, or even when I was working in Scotland, I worked in Edinburgh, and just say, even… some clients were obviously worried for themselves, but like, a lot of them really did not give a shit as well. I was just shocked, it was just like, like, I had only worked in Ireland and I had always been, like, stressed out a lot of time when I was working, and that I was gonna get discovered, and stuff like that, and then when I worked… in, in Australia and stuff like that, it was not like that at all, and that it was just like, it was just like this weight lifted off my shoulders, just like I’m, I’m ok, I can do this, and like, like, you feel like you’re not looking behind your shoulder and stuff’	‘You have to be very paranoid about where you live, I think that you know I’m gonna be outed, I’m gonna get kicked out again’ ‘…and so, I constantly have to be moving in and out of hotels and like, be worried about clients, and be worried about Gardaí’ ‘I think because you know people are going out of their way to harm you, you really pick up on that, on that energy, like people are actively trying to seek out where these, where these brothels are, and you’ll have to be kicked out, and that feels really intimidating’ ‘The fear of getting kicked out because people managed to successfully get me evicted, and threaten my kids and all that kind of stuff before, so I’ve gone through that and not had the, not had the police protection and fear of social services and all that, it’s like, it’s totally, it definitely feels completely different’
Discrimination	‘It’s something that you’re, you’re always aware of, you know, for example, you know a mom gave me a lift at school and then the next time she didn’t, she didn’t acknowledge me, or I messaged her, and she ignored me and I’m just like “oh has she found out I’m a sex worker,” so you’re always kind of cowering in that fear, especially when I’m around school’ ‘I’m feeling it there, I’m different, I’m a dirty secret, I’m, you, you know, are people looking at me and thinking that? So, it’s sorta, you definitely get para, paranoid, and I’m thinking my paranoia is quite justifiable considering, you know, everything that I have experienced, so, yeah, paranoia…because of how people…act, or…treat you or whatever’	‘And the, yeah, the effect of that was huge. And also, not only the objective offense of that, but the way this has been portrayed in the media, and the general feeling like, regular people, the general people are feeling toward us the way the way we are portrayed, and the way we are talked about, in the media vehicles, we are, it affects the way people think of us so that feeling plays on you as well plays on feeling lower, playing on the fear in general of being outed. Because now you know for sure you don’t have people on your side, and it makes fighting for change much more difficult. So, the Nordic model affected every, every aspect of my life negatively. I can’t see one good thing that the thing that this model brought to us’

While all interviews were carried out post hoc, quotes are included here where participants reflect on their experiences of working both before and after 2017 changes to sex work legislation in the Republic of Ireland

## Discussion

Though it has been suggested that sex workers have been decriminalised (Latham, 2013) under 2017 changes to legislation, this is untrue as aspects of sex work such as working together remain criminalised while penalties and fines for working together have been increased. Client criminalisation does not appear to have delivered the harm reduction that was promised by those that fought to implement it (Charlton, 2012; Latham, 2013; Bacik, 2013). Rather, similarly to 1993 changes (O’Connor et al., 1996), 2017 changes appear to have exacerbated existing stresses for sex workers. There is little if any research that includes the voices of sex workers and yet finds in favour of criminalising various aspects of sex work. Considering the involvement of religious orders in changes to sex work legislation, changes should perhaps be viewed with suspicion in light of the Republic of Ireland’s history of state-sanctioned institutionalised misogyny (Smith, 2010; Smith et al., 2013). As workers are understood to engage in sex work as a ‘result of deep structural inequalities, poverty and the lack of agency’ (Kelleher et al., 2009), it makes little sense to penalise various aspects of sex work and yet hope to somehow benefit workers, especially without having addressed the reasons a person might enter sex work in the first place. In 2019, a high-level working group that included representatives from the HSE (Health Service Executive), the Gardaí (police) and non-governmental organisations that spearheaded the move for client criminalisation such as Ruhama and the Immigrant Council of Ireland published a report (Shannon, 2019) looking at the effectiveness of the law to date. In the report, Ruhama are said to have found that 90% of women they encounter want to exit sex work but perceive that there are no viable alternatives for them. The report lists a number of barriers to exiting sex work such as poverty, a lack of viable alternatives, immigration status, homelessness and precarious housing, a lack of formal educational qualifications, psychological trauma, post-traumatic stress disorder and isolation. Sex work legislation in the Republic of Ireland contributes to isolation through brothel keeping laws which keep sex workers from working together in addition to contributing to homelessness for sex workers and precarious housing through eviction and prohibitions against renting to sex workers. Laws targeting migrants who engage in sex work contribute to poverty, jeopardise immigration status and are likely to contribute to psychological trauma and post-traumatic stress disorder. Parties responsible for sex work legislation in the Republic of Ireland appear to have contributed to a cycle of desperation with little opportunity for escape for those unfortunate enough to find themselves relying on sex work to support themselves and their families.

That the theme of psychological wellbeing arose more than any other, 3 years after the implementation of client criminalisation laws in Ireland is telling. Most workers spoke of the toll stigma and shame on themselves and their relationships, while all workers extolled the positive effects of the sex workers community. Previous research (O’Connor et al., 1996; Sanders, 2004) is supported with most participants expressing that the stress of living a double life negatively impacts their mental health. Suggestions by Latham (2013) during debate in the Oireachtas that sex workers in Ireland would no longer have to be covert under client criminalisation has not only failed to come to fruition but was unrealistic considering the Republic of Ireland’s history of institutional misogyny. Findings by Huschke and Ward (2017) and Ellison et al. (2019) were supported with workers feeling that they had little choice other than to drop prices and let their boundaries be eroded.

No participants spoke in positive terms of law enforcement in Ireland. The findings of this study supported that of previous research (Valiulis et al., 2007; Lutnick & Cohan, 2009; Huschke & Ward, 2017; Platt et al., 2018; Ellison et al., 2019) with participants expressing trepidation in relation to contact with law enforcement. Findings did not support Latham’s (2013) or Pringle’s (2013) supposition that workers would feel encouraged to report violence, robberies and rape without fear of retribution or incriminating themselves. While more than one participant expressed that they might not contact Gardaí (police) even in a life-or-death situation, one participant declined to contact them for help even after they had been raped. Until such time as a workable relationship between sex workers and Gardaí can be achieved and trust established, there is a demand for alternative sources of assistance for sex workers in need.

Rachel’s suggestion that health checks be carried out by health officials rather than Gardaí that have the power to evict, jail and deport sex workers is one such solution. As it has been repeatedly stated by influential members of Irish society instrumental in the implementation of changes to sex work legislation in the Republic of Ireland that harm reduction to sex workers is the intended goal, it is worth considering that current approaches are not working. Alternative models such as decriminalisation that are employed in New Zealand and New South Wales, while not without their flaws, are worth serious consideration. Sex workers have been shut out of decisions that directly affect them. It is vital that their voices are included in decision-making going forward.

## Future Research

The current study examines how sex workers in Ireland experience laws that criminalise various aspects of the exchange while increasing fines and sentences for others. It would be beneficial to repeat this study with a larger sample and perform similar studies with sex workers, including undocumented sex workers, living and working under decriminalisation in New Zealand or New South Wales for comparison.

## Strengths and Limitations

A major strength of this research was the willingness of sex workers in Ireland to contribute and have their voices, opinions and perspectives heard. The researcher had access to a diverse sample of sex workers from diverse countries of origin, circumstances and entry points into sex work. This piece of research explores perceptions of a piece of legislation by those most affected by it. The age range and types of sex work engaged in were broad and representative considering the small sample size. Originality is a strength of this work as there are few studies on sex work employing IPA. Limitations include the small sample size. Climates that are hostile to sex workers likely contribute to invisibility amongst this almost invisible demographic, creating a catch-22 situation for researchers. Mental health is measured by self-reporting rather than validated measurement tools. This is a post hoc study.

## Conclusion

Client criminalisation does not appear to have improved life for sex workers in Ireland. Instead, changes to the law appear to have contributed to a climate of anti-sex worker hostility, exacerbating existing stresses, such as discrimination and fear of eviction. Already tense relationships with law enforcement appear to have worsened under present legislation. As suggested by participants, it is worth exploring whether services such as welfare checks should be performed by parties other than law enforcement. Research is needed to explore whether decriminalisation might provide some solutions to problems client criminalisation was intended to address, such as willingness for sex workers to contact and cooperate with law enforcement without fear of repercussions (Latham, 2013). Decriminalisation could perhaps serve as a step toward reducing stigma which would benefit the mental health of sex workers. A reduction in shame and stigma may benefit relationships with friends and family and in dealings with healthcare workers and service providers.
